# Acute renal failure, COVID-19 and deaths, worrying rates in intensive care units: a cross-sectional study

**DOI:** 10.1590/1516-3180.2023.0150.R1.13052024

**Published:** 2024-08-09

**Authors:** Yoshimi José Ávila Watanabe, Lívia Maria Rezende Carvalho, João Victor Marques Guedes, André Oliveira Baldoni, Vinícius Silva Belo, Alba Otoni

**Affiliations:** I Nefrologist, Physician, PhD Student, Postgraduate Program in Health Sciences, Campus Centro Oeste (CCO), Universidade Federal de São João del-Rei (UFSJ), Divinópolis (MG), Brazil.; II Undergraduate Nursing Student, Curso de Enfermagem, Campus Centro Oeste (CCO), Universidade Federal de São João del-Rei (UFSJ), Divinópolis (MG), Brazil.; III Pharmacist, External Research Partner, Postgraduate Program in Health Sciences Campus Centro Oeste (CCO), Universidade Federal de São João del-Rei (UFSJ), Divinópolis (MG), Brazil.; IV Pharmacist, Associate Professor, Campus Centro Oeste (CCO), Universidade Federal de São João del-Rei (UFSJ), Divinópolis (MG), Brazil.; V Biologist, Associate Professor, Campus Centro Oeste (CCO), Universidade Federal de São João del-Rei (UFSJ), Divinópolis (MG), Brazil.; VI Nurse, Associate Professor, Campus Centro Oeste (CCO), Universidade Federal de São João del-Rei (UFSJ), Divinópolis (MG), Brazil.

**Keywords:** Acute kidney injury., Death., Intensive care units., COVID-19., Kidney damage., Intensive therapy., Viral infection., Pandemic.

## Abstract

**BACKGROUND:**

Acute kidney failure is a serious consequence of coronavirus disease 2019 (COVID-19).

**OBJECTIVES:**

To identify the prevalence of COVID-19, kidney failure, frequency of death, and associated factors in patients receiving intensive care.

**DESIGN AND SETTING:**

Analytical cross-sectional study conducted in the intensive care unit (ICU) of a medium-sized philanthropic general hospital in center-west Minas Gerais.

**METHODS:**

Adults and older individuals who underwent real-time polymerase chain reaction testing for severe acute respiratory syndrome coronavirus 2 (SARS-CoV-2) were evaluated by the nephrology team.

**RESULTS:**

Among the 176 patients, the prevalence of COVID-19 and acute kidney injury (AKI) were 103 (58.5%) and 132 (75%), respectively, and 44 (25%) had chronic kidney disease (CKD) and 16 (15,5%) were positive for SARS-CoV-2. In the Charlson index classification, which estimates the risk of death, a statistically significant difference was identified in the percentages of groups with and without COVID-19 for indices 0, 1, and 2. There was a significant association between kidney disease and ICU mortality (P < 0.05). Patients with CKD had fewer fatal outcomes (13/97, 13.4%) than those with AKI (85/97, 87.6%).

**CONCLUSIONS:**

COVID-19 rates remained high long after diagnosis and prevention of SARS-CoV-2 infection. In addition, a higher death rate among patients who developed AKI, whose prevalence was also greater than that in the national literature, regardless of the presence of COVID-19, revealed a worrying scenario and corroborated the need for early and judicious approaches to preserve the lives of patients with AKI admitted to intensive care units.

## INTRODUCTION

Acute kidney failure is a serious consequence of severe acute respiratory syndrome coronavirus 2 (SARS-CoV-2) infection. Its occurrence can vary from 0.5% to 7% of the general population, being more frequent among those who are hospitalized and mainly in those who require intensive treatment.^1,2^ During the coronavirus disease 2019 (COVID-19) pandemic, a greater number of patients with acute kidney injury (AKI) were observed in Brazil and worldwide.^3,4,5^

Specifically in Brazil, a study from Rio de Janeiro reported a high incidence of AKI in individuals with COVID-19 and an association with high mortality, highlighting the risk factors or predictors: acute respiratory distress syndrome, age, altered glomerular filtration rate, and systemic arterial hypertension.^6^ In addition to AKI, the mortality rate was high for patients with chronic kidney disease (CKD), understood as abnormalities in the structure and/or function of the kidneys present for more than three months with implications for health, in Brazil.^7^ The authors suggested that comorbid events, socioeconomic conditions, health system deficiencies, structural care inefficiencies, as well as social inequality allowed the advance of this pandemic infectious disease with a high frequency of lethal outcomes.^7^

People infected with SARS-CoV-2 have different outcomes, particularly when comparing those with and without known prior kidney disease. The reasons suggested for the different courses and outcomes are still inconclusive but suggest that there is an association with inflammatory “status” and response to infection, as the main motivator and promoter of the outcomes.^6^

Considering the severity and lethal impact of COVID-19, it is important to understand the epidemiological profile of patients affected by the disease and those with dysfunction or metabolic disorders involving the kidneys. Such information will be useful to better understand the evolutionary course of this group of patients and, consequently, enable a better propaedeutic and therapeutic approach as well as logistical, financial, and operational management of the public health system.

## OBJECTIVE

This study aimed to identify the prevalence of COVID-19, kidney failure, frequency of death, and associated factors in patients admitted to an intensive care unit (ICU).

## METHODS

### Outline

This cross-sectional study was conducted during the first half of 2022 in the intensive care unit of a large hospital complex in the Midwest region of Minas Gerais, Brazil. The Strengthening the Reporting of Observational Studies in Epidemiology (STROBE) guidelines were adopted to strengthen and guarantee the requirements of an observational study.^8^ The Nephrology Service was activated to evaluate patients with renal alteration or metabolic dysfunction, with hospitalization ranging from zero to four days for those without COVID-19 (Tested Negative for SARS-CoV-2) and one to 11 days for the COVID-19 group (Tested Positive for SARS-CoV-2). Medical records were used for evaluation.

### Participants and sample power calculation

Adult and older patients of both sexes who were admitted to the ICU in the first half of 2022 with evidence of renal impairment, which is subsequently referred to as kidney failure, and who underwent the real-time polymerase chain reaction (RT-PCR) test for SARS-CoV-2, were considered eligible, and those with incomplete information in their medical records were excluded.

**Figure 1:**
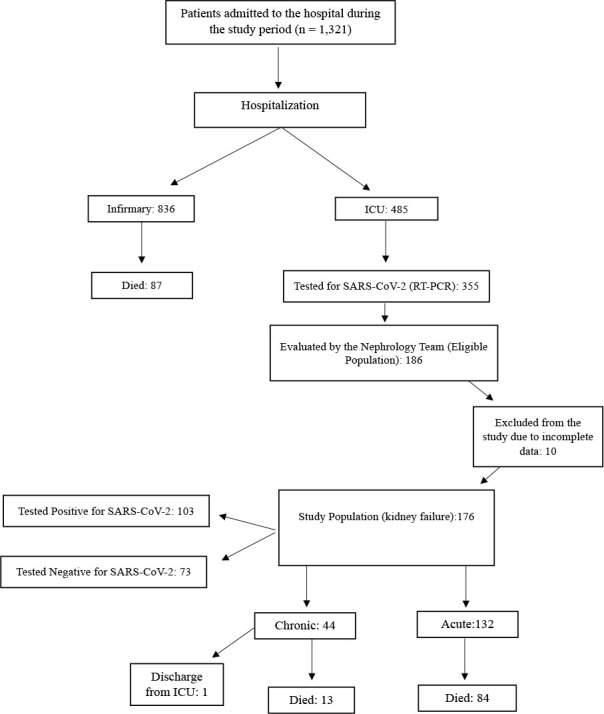
Flowchart of patients evaluated in the study and evolution to death.

ICU, intensive care unit; SARS-CoV-2, severe acute respiratory syndrome coronavirus 2 2019; RT-PCR, real-time reverse transcription polymerase chain reaction.

During the study period, 1,321 patients were hospitalized. Of these, 485 were admitted to the ICU and 836 were admitted to the infirmary, of whom 87 died. Among those admitted to the IUC (485), 355 underwent tests for SARS-Cov-2, and 186 also had kidney failure. Therefore, this cohort was eligible for inclusion in this study. Of these, 10 were excluded because of incomplete data, hence the study cohort comprised 176 participants. Of these, 103 tested positive, and 73 tested negative for SARS-CoV-2. Among the same cohort of 176 patients who had kidney failure, 132 had AKI and 44 had CKD. Among those with CKD, one was discharged from the ICU and 13 died. It is noteworthy that only for the analysis of mortality, the patient with CKD who was discharged from the ICU was not included in this analysis (**Figure 1**).

The estimation of sample size was estimated using G*Power software, version 3.1.9.2. (Written by Franz Paul, University of Kiel, Kiel, Germany). The prevalence of kidney disease in patients without COVID-19 (73) and with COVID-19 (103) was considered at a significance level of 5% and the sample power was 92.7%.

### Study variables of interest

The following outcomes were considered: the results of the SAR-Cov-2 test (positive or negative) using RT-PCR. In addition, the outcome (fatal or nonfatal) was considered to assess the impact of kidney failure on death rates, with the presence or absence of CKD and AKI as the main explanatory variables, in addition to the other explanatory variables for both outcomes, as described below.

The explanatory variables were grouped into sociodemographic: age (continuous) and sex; and clinical: present or absent comorbidities; presence of Stage 3 acute kidney disease classified as creatinine ≥ 4.0 mg/dL or ≥ 3 times baseline, or decline in urine output by < 0.3 mL/kg/h for ≥ 24 h, or anuric for ≥ 12 h, and presence of CKD;^9^ renal replacement therapy; and the Charlson Comorbidity Index, which uses 19 diseases that have a fixed weight assigned that vary between one and six points based on the severity of the condition.^10^

### Statistical analysis

Frequency and dispersion measures were used to characterize the sociodemographic and clinical profiles of the cohort. Categorical variables are presented as absolute values and percentages and quantitative variables as medians and quartiles, considering the asymmetric distribution of data, as indicated by the Shapiro-Wilk test.

Associations between sociodemographic and clinical variables and outcomes were analyzed using the Chi-square test or Fisher’s Exact test. The association between quantitative variables and the outcomes was determined using the Mann–Whitney U test. For multiple analysis in the logistic regression models, all explanatory variables that were P < 0.20 in the bivariate association were included, and the backward technique was applied. For the final model, as well as for the statistical tests conducted, a significance level of P < 0.05 was adopted. All analyses were performed using Statistical Package for the Social Sciences, version 25 (SPSS, IBM, Armonk, New York, United States).

### Ethical aspects

This study was approved by the Ethics Committee for Research involving Human Beings of the Universidade Federal de São João del-Rei (C.A.A.E. 11780919.8.0000.5545) with technical approval number: 3,359,799, on July 18, 2019. To guarantee the consent of the participants, an Informed Consent Form was presented to each patient, which was then signed.

## RESULTS

Of the total participants, the majority were male (97 = 55.1%) and older with a median age of 60 years (Quartiles: 47-73). The frequencies of COVID-19, AKI, and CKD were, 103 (58.5%) 132 (75%), and 44 (25%), respectively. There were 73 (41,4%) patients without COVID-19. The frequency of AKI was significantly higher in patients with COVID-19 (**Table 1**).

**Table 1:** Association between the presence and absence of coronavirus disease 2019 with sociodemographic and clinical variables in patients evaluated by nephrology, admitted to the intensive care unit of a large hospital in a city in the Midwest of Minas Gerais in the first half of 2022 (n = 176)

Variables	COVID-19	COVID-19	Total	P*
	negative	positive		
	(n = 73)	(n = 103)		
**Sex**				
Female	34 (46.6%)	45 (43.7%)	79 (44.9%)	0.70
Male	39 (53.4%)	58 (56.3%)	97 (55.1%)	
**Age** (median)	63 (55 – 73)	60 (47 – 71)		0.28
**Cardiopathy**				
No	54 (74.0%)	89 (86.4%)	143 (81.3%)	0.03
Yes	19 (26.0%)	14 (13.6%)	33 (18.8%)	
**Vasculopathy**				
No	63 (86.3%)	99 (96.1%)	162 (92.0%)	0.02
Yes	10 (13.7%)	4 (3.9%)	14 (8.0%)	
**DM**				
No	52 (71.2%)	78 (75.7%)	130 (73.9%)	0.50
Yes	21 (28.8%)	25 (24.3%)	46 (26.1%)	
**SAH**				
No	50 (68.5%)	69 (67.0%)	119 (67.6%)	0.83
Yes	23 (31.5%)	34 (33.0%)	57 (32.4%)	
**Pneumopathy**				
No	71 (97.3%)	91 (88.3%)	162 (92.0%)	0.04
Yes	2 (2.7%)	12 (11.7%)	14 (8.0%)	
**Oncology disorders**				
No	69 (94.5%)	103 (100.0%)	172 (97.7%)	0.02
Yes	4 (5.5%)	0 (0%)	4 (2.3%)	
**Kidney disease**				
Acute	45 (61.6%)	87 (84.5%)	132 (75.0%)	<0.01
Chronic	28 (38.4%)	16 (15.5%)	44 (25.0%)	
**Charlson index**				
0**	30 (41.1%)	60 (58.3%)	90 (51.1%)	0.04
1 – 2**	20 (27.4%)	12 (11.7%)	32 (18.2%)	
3 – 4	12 (16.4%)	16 (15.5%)	28 (15.9%)	
≥ 5	11 (15.1%)	15 (14.6%)	26 (14.8%)	
**Obesity (n = 109)**				
No	46 (93.9%)	47 (78.3%)	93 (85.3%)	0.02
Yes	3 (6.1%)	13 (21.7%)	16 (14.7%)	
**ICU Death (n = 176)**				
No	43 (58.9%)	35 (34.3%)	78 (44.6%)	<0.01
Yes	30 (41.1%)	67 (65.7%)	97 (55.4%)	
**RRT (n = 119)**				
No	34 (65.4%)	30 (44.8%)	64 (53.8%)	0.02
Yes	18 (34.6%)	37 (55.2%)	55 (46.2%)	

*Chi Square Test and Fisher’s Test; **Statistically different values between COVID- and COVID+; ICU = intensive care unit; DM = diabetes mellitus; SAH = systemic arterial hypertension; RRT = Renal Replacement Therapy.

In the Charlson Comorbidity Index, a statistically significant difference was identified between the COVID-19 group and the non-COVID-19 group in the proportions of individuals with indices 0, 1, and 2, which are considered lighter indices. There was a higher proportion of the zero index in the COVID-19 group and indices 1-2 had higher proportions in the non-COVID-19 group.

The bivariate analysis of the association between COVID-19 outcomes and explanatory variables is shown in **Table 1**.

With regard to the multivariate analysis, the variables with P < 0.20 and thus included in the model, were comorbidities (heart disease, vasculopathy, pneumopathy, oncology disorders, and obesity), kidney failure, Charlson Comorbidity Index, death, and renal replacement therapy. However, in the final model, none showed a significant association with the COVID-19 and non-COVID-19 outcomes.

When performing the bivariate analysis considering death as the outcome, a significant association was identified in the progression to death between patients with AKI and those with CKD (P = .01). Patients with CKD had a lower mortality rate (13/97, 13.4%) compared with those diagnosed with AKI (85/97, 87.6%) (**Table 2**). However, this association was not significant in the multivariate logistic regression. When patients simultaneously had COVID-19 and AKI (n = 87), the mortality rate was 69.0% (data not shown).

**Table 2:** Association between the presence and absence of death in the intensive care unit with sociodemographic and clinical variables in patients evaluated by nephrology, admitted to the intensive care unit of a large hospital in a city in the Midwest of Minas Gerais in the first half of 2022 (n = 175)

Variables	No	Yes	Total	P*
	(n = 78)	(n = 97)		
**Sex**				
Female	31 (39.7%)	48 (49.5%)	79 (45.1%)	0.19
Male	47 (60.3%)	49 (50.5%)	96 (54.9%)	
**Age** (median)	61 (49 – 72)	62 (48 – 70)		0.20
**Cardiopathy**				
No	63 (80.8%)	79 (81.4%)	142 (81.1%)	0.91
Yes	15 (19.2%)	18 (18.6%)	33 (18.9%)	
**Vasculopathy**				
No	71 (91%)	90 (92.8%)	161 (92.0%)	0.67
Yes	7 (9%)	7 (7.2%)	14 (8.0%)	
**DM**				
No	55 (70.5%)	74 (76.3%)	129 (73.7%)	0.38
Yes	23 (29.5%)	23 (23.7%)	46 (26.3%)	
**SAH**				
No	53 (67.9%)	65 (67.0%)	118 (67.4%)	0.89
Yes	25 (32.1%)	32 (33.0%)	57 (32.6%)	
**Pneumopathy**				
No	73 (93.6%)	88 (90.7%)	161 (92.0%)	0.48
Yes	5 (6.4%)	9 (9.3%)	14 (8.0%)	
**Oncology disorders**				
No	76 (97.4%)	95 (97.9%)	171 (97.7%)	0.99
Yes	2 (2.6%)	2 (2.1%)	4 (2.3%)	
**Kidney disease**				
Acute	48 (61.5%)	84 (87.6%)	132 (75.4%)	<0.01
Chronic	30 (38.5%)	13 (13.4%)	43 (24.6%)	
**Charlson index**				
0**	32 (41%)	58 (59.8%)	90 (51.4%)	0.01
1 – 2**	21 (26.9%)	10 (10.3%)	31 (17.7%)	
3 – 4	11 (14.1%)	17 (17.5%)	28 (16.0%)	
≥ 5	14 (17.9%)	12 (12.4%)	26 (14.9%)	
**Obesity (n = 109)**				
No	40 (93%)	53 (80.3%)	93 (85.3%)	0.09
Yes	3 (7%)	13 (19.7%)	16 (14.7%)	
**COVID (n = 175)**				
Negative	43 (55.1%)	30 (30.9%)	73 (41.7%)	<0,01
Positive	35 (44.9%)	67 (69.1%)	102 (58.3%)	
**RRT (n = 119)**				
No	24 (48%)	40 (58%)	64 (53.8%)	0.28
Yes	26 (52%)	29 (42%)	55 (46.2%)	

* Chi Square Test and Fisher’s Test; **Statistically different values between death and non-death in the ICU only in the marked classes; DM = diabetes mellitus; SAH = systemic arterial hypertension; RRT = Renal Replacement Therapy.

## DISCUSSION

The increased frequency of COVID-19 with simultaneous AKI in the ICU has revealed a worrying scenario. During the second wave of COVID-19 in Brazil, in which many severe cases were recorded, acute renal involvement was to be expected among people hospitalized with the SARS-CoV-2 virus but the prevalence rate found in this study was higher than the rates of AKI described in the current literature, which had a mean of approximately 60%.^5,12,13^

In a hospital study performed in China, whose objective was to evaluate the impact of AKI on the clinical evolution of COVID-19, an incidence of 12.9% of AKI was identified, increasing the risk of death for those hospitalized for COVID-19.^14^ Corroborating these findings, a cohort developed in New York with 5,449 people identified that 1,993 (36.6%) developed AKI, 31.1% of these in Stage 3.^15^

In the Brazil, the percentage of patients with AKI has been variable, but still below that found in the present study. In a study performed in the state of Paraná/Brazil, it was found that 11.6% of patients developed AKI.^16^ In contrast, a study performed in the state of Rio de Janeiro reported that 55.9% of patients with COVID-19 admitted to the ICU developed AKI, with the majority (66.7%) advancing to Stage 3.^4^

In view of the results of national surveys, it is necessary to reflect on the reason for such high rates of AKI in the patients involved in the current study, as well as the reason for the wide variety of AKI frequencies of acute kidney disease in ICUs. Such diverse perspectives can be explained by population demographics, comorbidities, and disease severity inherent to each patient profile and Brazilian regional location.

With regard to the pandemic period, many other factors could account for the high prevalence of AKI triggered by COVID-19, including the hyperinflammatory state due to the virus binding to angiotensin-converting enzyme 2 receptors, perfusion deficits, and the use of nephrotoxic drugs, which may favor tubulointerstitial nephritis and affect the kidney as a whole.^6^ It is important to note that there is a consensus in the literature that the development of AKI in patients with COVID-19 decreases their survival and exponentially increases the risk of death.^4,17,18^

This consensus from the literature was corroborated by our findings, which, demonstrated the impact on the mortality of patients with COVID-19 and simultaneous AKI and revealed a mortality rate of almost 70%, higher than that reported in the literature.

Hirsch et al.^15^ identified a 35% mortality rate among patients with COVID-19 and AKI and showed that mortality was higher among those classified as having Stage 3 acute renal impairment. In a study by Costa et al.^4^ a significant difference was found in the mortality rates between patients with COVID-19 with and without renal involvement (33.3% and 9%, respectively). In another important study, Aroca et al.^17^ reported that patients with COVID-19 who developed AKI had an 11.83 times greater risk of death than those without AKI.

It is also interesting to note that, although not common in national studies, international studies have emphasized the Charlson Comorbidity Index to weigh the risk of death and add more solidity to the results related to mortality rates.^11^ In this perspective, although the multivariate analysis of this study did not find significant associations between the Charlson Comorbidity Index and the presence of COVID-19 or between the Charlson Comorbidity Index and death, the consensus view in the literature is that most patients diagnosed with COVID-19 and comorbidities, most commonly heart disease, systemic arterial hypertension, diabetes mellitus, and obesity, have a higher mortality rate.^4,16,18,19,20^

Finally, considering the entire scenario presented based on the literature and aligned with our results, we can state that the evolution of COVID-19 combined with renal dysfunction contributes to the aggravation of the patient’s condition, which may explain the failure of multiple organs and high death rate in this group.

### Study limitations

The results of this study cannot be generalized because they were conducted on individuals admitted to a single hospital. It should be noted that the lack of official health records of information essential for conducting the study limited the inclusion of more participants. Brazil is still not effectively using official health records as a secondary and complete source of data. Most national studies point to the lack of data as a limiting factor for achieving a larger “n” and for more complex analyses.

Even so, it should be noted that the institution where the study was carried out is a reference hospital for COVID-19 for the population of 54 municipalities, which together represent approximately one million people, and therefore increases the representativeness of the results shown here.

## CONCLUSION

This study revealed a high rate of COVID 19 simultaneously with a high rate of AKI, demonstrating that even after more than two and a half years of the pandemic there is still much to understand regarding the main measures to prevent complications, associated comorbidities, and effective approaches to this virus. The mortality rate among patients with AKI was also above expectations (87.6%), maintaining a rate of almost 70% in the presence of COVID-19. It is recommended that further studies with a longitudinal design and with a larger sample size be performed to determine, with certainty, solid evidence that explains the causes of such high rates of complications and mortality associated with AKI, as well as with COVID-19.
